# Advocacy and Raising Awareness for Atypical AD

**DOI:** 10.1002/alz70860_098421

**Published:** 2025-12-23

**Authors:** CHUKWUANUGO OGBUAGU

**Affiliations:** ^1^ Nnamdi Azikiwe University Teaching Hospital (NAUTH), Nnewi, Nigeria; Global Brain Health Institute, University of California, San Francisco, CA, USA

## Abstract

Atypical forms of Alzheimer's Disease (AD) usually have specific methods of diagnosis and pose occasional challenges because of their non‐traditional features of presentations, which most times delay diagnosis. These manifestations lead to improper classification when compared with the common forms of Alzheimer's. The uncommon types of ADs most often affect vision, language, or movement early in the disease stage before memory deficits become prominent, thereby complicating early diagnosis. This manuscript highlights the importance of advocacy and awareness in tackling these challenges while focusing on public health education for healthcare providers, patients, caregivers, and the public about these uncommon presentations.

We propose that with a targeted approach and intentional campaign and training program, we aim to improve early detection, reduce stigma, enhance access to specialized care, and prime clinical trials for individuals with atypical Alzheimer's. Additionally, we emphasize the need for research equity to further trials and investment in diagnostic tools and treatment pathways tailored to these variants. By fostering collaboration between policymakers, healthcare systems, and communities, this advocacy effort seeks to bridge knowledge gaps and improve outcomes for those affected by atypical Alzheimer's disease globally.

Advocacy and raising awareness of atypical AD through the inclusion of diverse populations in global clinical research are needed. This is essential to improving early diagnosis, developing targeted global treatments, and addressing disparities in care. Ultimately, this will ensure that patients with atypical presentations from different backgrounds receive the recognition and support they need.

